# The effect of urinary essential and non-essential elements on serum albumin: Evidence from a community-based study of the elderly in Beijing

**DOI:** 10.3389/fnut.2022.946245

**Published:** 2022-07-18

**Authors:** Ang Li, Quan Zhou, Yayuan Mei, Jiaxin Zhao, Liu Liu, Meiduo Zhao, Jing Xu, Xiaoyu Ge, Qun Xu

**Affiliations:** ^1^Department of Epidemiology and Biostatistics, School of Basic Medicine Peking Union Medical College, Institute of Basic Medical Sciences Chinese Academy of Medical Sciences, Beijing, China; ^2^Center of Environmental and Health Sciences, Peking Union Medical College, Chinese Academy of Medical Sciences, Beijing, China; ^3^Chaoyang District Center for Disease Control and Prevention, Beijing, China

**Keywords:** serum albumin, nutritional status, bayesian kernel machine regression, quantile g-computation, elements

## Abstract

**Background & aims:**

Few epidemiological studies have investigated the relationships of urinary essential and non-essential elements with serum albumin, an indicator of nutritional status, especially for the elderly in China.

**Methods:**

A community-based study among elderly participants (*n* = 275) was conducted in Beijing from November to December 2016. We measured 15 urinary elements concentrations and serum albumin levels. Three statistical methods including the generalized linear model (GLM), quantile g-computation model (qgcomp) and bayesian kernel machine regression (BKMR) were adapted.

**Results:**

In GLM analysis, we observed decreased serum albumin levels associated with elevated urinary concentrations of aluminum, arsenic, barium, cobalt, chromium, copper, iron, manganese, selenium, strontium, and zinc. Compared with the lowest tertile, the highest tertile of cadmium and cesium was also negatively associated with serum albumin. Urinary selenium concentration had the most significant negative contribution (30.05%) in the qgcomp analysis. The negative correlations of element mixtures with serum albumin were also observed in BKMR analysis.

**Conclusions:**

Our findings suggested the negative associations of essential and non-essential elements with serum albumin among the elderly. Large-scare cohort studies among the general population are required to validate our findings and elucidate the relevant underlying mechanisms.

## Introduction

In 2020, persons aged 65 years and older were estimated to be 727 million worldwide and projected to be over 1.5 billion by 2050 (1). The elderly are particularly at risk of malnutrition due to impaired metabolism and organic function and declined physiological reserves during the aging process (2–4). Common signs of malnutrition include unexpected weight loss and fatigue (5). The estimated prevalence of malnutrition in the elderly living in the community ranges between 7.8 and 45.4%, which differs by country (6, 7). Malnutrition furthermore triggers elevated cardiovascular disease risk and excess mortality (8, 9). As a significant transport protein synthesized in the liver, serum albumin was widely utilized to assess the nutritional state and diagnose malnutrition (10). In order to prevent and intervene against malnutrition in the elderly, it is necessary to identify the potential risk factors and understand the mechanisms of malnutrition. The high prevalence of malnutrition among the elderly may be partly due to global aging. However, it still cannot be explained by traditional risk factors such as social driving factors and ecological factors (11).

Essential and non-essential elements were absorbed into the body mainly through diet (12). Heavy metals have a long half-life, and ongoing exposure leads to their bioaccumulation in the body[e.g., cadmium (Cd) has a half-life of 10–30 years] (13, 14). A disturbance in the delicate balance of essential nutrients can also negatively impact health (15). Consequently, essential and non-essential elements were linked with many adverse health effects, including low serum albumin levels (16–18). Experimental studies have suggested that metal ions, including cobalt (Co), chromium (Cr), and nickel (Ni) induce aggregation synergistically of human serum albumin (19). Furthermore, serum albumin levels in Wistar rats decreased after being exposed to 100 times the mode concentrations of the each element in the mixtures of elements [arsenic (As), Cd, lead (Pb), mercury, Cr, Ni, manganese (Mn), and iron (Fe)] *via* drinking water for 90 days (20). Epidemiological studies have discovered that elements were associated with serum albumin. In detail, a cross-sectional study of 240 male tannery workers reported that prolonged exposure to hexavalent Cr (Mean: 4.89 μg/L in smokers) is likely to reduce serum albumin levels (21).

Although studies have begun to pay attention to the relationship between urinary essential and non-essential elements, and serum albumin, several knowledge gaps in this field must be bridged. First, essential and non-essential elements are often released into the environment (e.g., air, water, soil) as mixtures. Individuals are exposed to multiple elements simultaneously (22). However, epidemiological studies, especially in exploring the effect of the element mixtures on serum albumin, are still limited in the current literature. Second, the traditional generalized linear model (GLM) was broadly used to estimate associations of elements with health outcomes based on the hypothesis that the associations were linear. However, non-additive and non-linear effects of elements on health under real-world scenarios should be considered (23, 24). Bayesian kernel machine regression (BKMR) models were recommended to investigate the combined effects of mixtures. Furthermore, the quantile based g-computation (qgcomp) approach was also utilized, considering residual confounding, and uncertain effect direction of elements in the mixtures (25). At the same time, few studies have simultaneously adopted these methods to explore associations between the element mixtures and serum albumin in the real-world scenario.

In a cross-sectional study of 275 elderly participants residing in Beijing, China, in November and December 2016, we utilized three different statistical methods to explore associations of essential and non-essential elements with serum albumin. The results of this study will provide information on the effects of these elements on the serum albumin.

## Methods

### Study setting and population

The study was conducted in communities distributed from south to north in Beijing, which experienced the highest levels of fine particulate matter during the last 20 years in China (26). Further details regarding the study setting and design as well as inclusion and exclusion criteria were previously mentioned (27, 28). Eligible participants were the elderly (above 60 years or more) who lived in the local community for over 5 years. We excluded subjects who were unable to complete anthropometric examinations or questionnaire surveys. Furthermore, we excluded participants who had the malignant tumor, cardiovascular event, liver disease, or endocrine disease. Participants who refused to provide biological samples were further excluded. A total of 275 subjects were finally included in the analyses. The study was approved by the institutional ethics committees of the Institute of Basic Medicine in the Chinese Academy of Medical Sciences. Written informed consents were obtained from all participants.

### Measurements of samples

The participants fasted overnight (≥8 h) before examinations. Peripheral blood samples (4 mL) were obtained from all participants by the qualified nurses. First-morning urine samples were collected from the participants in trace element–free containers. All urine and blood samples were stored at −80°C for subsequent analysis.

The inductively coupled plasma mass spectrometry (Nexion 300D, PerkinElmer SCIEX, USA) was utilized to analyze urinary samples. The detected elements included aluminum (Al), Cr, Mn, Fe, Co, Ni, copper (Cu), zinc (Zn), As, selenium (Se), strontium (Sr), Cd, cesium (Cs), barium (Ba), and Pb. Before analysis, one milliliter of urine was made up to 15 mL with 0.5% (v/v) HNO_3_ and 0.02% Triton X-100 and treated by sonication in an ultrasonic water bath for 1 h at 60°C. In addition, we applied quality control protocols to ensure the accuracy of our analyses. First, all the samples were measured three times, and the mean was calculated for subsequent analysis. Second, standard reference materials were measured every 20 samples to ensure the correct results of the studied specimens. The values below the limit of detection (LOD) were assigned LOD/2 for subsequent statistic analysis. The concentrations of urinary elements were all corrected by urinary creatinine to control for the effect of urine dilution. Creatinine-adjusted urinary elements concentrations were utilized in subsequent analyses. Urinary creatinine concentrations were measured on a Beckman Coulter analyzer (AU5800 Analyzer, Beckman Coulter, Brea, CA, USA).

The serum samples were sent to the Clinical Laboratory Center of Peking Union Medical College Hospital for clinical laboratory examinations. Serum albumin, glucose, and high-sensitivity C-reactive protein (hs-CRP) levels were measured using a Beckman Coulter analyser (AU2700, Beckman Coulter, Brea, CA, USA).

### Anthropometric measurements

The participants' upper arm blood pressure was measured using calibrated mercury sphygmomanometer at least three times after the participants had rested for 15 min in a room. Systolic blood pressure and diastolic blood pressure was obtained at the appearance and disappearance of Korotkoff sounds, respectively. The interval between measurements was required to exceed 2 min. Additional measurements were taken if the differences among measurements exceeded 5 mmHg. The means of the final two measurements were calculated for subsequent analysis. Height was measured using a verified stadiometer, with each participant in a standing position with shoes removed, shoulders relaxed, head facing forwards, and back facing the wall. Weight was measured with the participants wearing little clothing by using a certified body composition analyzer. The participants' weights in kilograms were divided by their heights in meters squared to obtain their body mass index (BMI). The measurements of height and weight were performed twice and averaged for reliability and accuracy (29).

### Ascertainment of covariates

Questionnaires were used to obtain the demographic characteristics, including age (years), sex (male or female), educational attainment (primary school or below, middle school, high school, or above); lifestyle characteristics, such as cigarette smoking (current, former, and never) and alcohol consumption (current, former, and never) habits; and health status, including hypertension (yes/no) and diabetes (yes/no). Participants who had smoked at least a cigarette per day for more than 6 months were defined as current smokers (30), and participants who had drunk alcohol at least once per week for more than 6 months were considered current drinkers (31). Hypertension was indicated by any antihypertensive medication prescription or blood pressures ≥140/90 mmHg measured under standardized clinical conditions (32). Diabetes was defined as fasting glucose ≥7.0 mmol/L, 2-h glucose ≥11.1 mmol/L, or current antidiabetic medication use (both insulin or oral antidiabetic drugs) (33).

### Statistical analysis

#### Statistical description

The general characteristics, demographic features, and clinical features of participants were described. Mean and standard deviation (SD) were used to describe normal distribution data, and the interquartile range (IQR) and median were used to describe data with skewness distribution.The categorical variables are expressed as number (percentage). All skewed distribution variables were logarithmically converted into approximately normal distributions for subsequent analysis.

#### GLM analysis

Associations between urinary essential and non-essential elements and serum albumin were assessed using the GLM. The GLM could estimate linear associations with following the assumptions like normality of outcome variables. First, we explore the associations between continuous urinary elements concentrations and serum albumin. The data were presented as regression coefficients and 95% confidence intervals (CIs) of changes in serum albumin associated with a unit increase in urinary elements concentrations. Second, the urinary elements concentrations were included in the models as categorical variables indicating tertiles, and the lowest tertile was the referent category. The regression coefficients and 95% CIs were also reported to indicate the association between urinary elements concentration and serum albumin in each tertile, respectively. Based on prior knowledge and literature accumulation, we adjusted age, sex, BMI, educational attainment, cigarette smoking habits, alcohol consumption habits, hypertension status, and diabetes status in the models. To address the multiple testing problem and reduce the probability of type I error, we adjusted the raw *p*-values on the basis of the false discovery rate by using Benjamini–Hochberg procedure (34).

#### Stratified analysis and sensitivity analysis

To account for effect modification of the GLM analysis, we conducted analyses stratified by demographic characteristics [age (<65 or ≥65 years) and sex (male or female)], and habits [cigarette smoking (yes or no) and alcohol consumption (yes or no)], while simultaneously controlling for the same covariates as in the GLM analysis. The differences between strata were examined by estimating values and 95% CIs as follows (35).


$$(\beta_{1}-\beta_{2})\;\pm \;1.96\sqrt{{\left(SE_{1}\right)}^{2}+{\left(SE_{2}\right)}^{2}}$$

Where β_1_ and β_2_ are the effect estimates attributed to each subgroup or stratum (e.g., the effects for participants with and without cigarette smoking, respectively), and *SE*_1_ and *SE*_2_ are the corresponding standard errors.

We conducted sensitivity analyses to assess the robustness of the findings in the GLM analysis. The hs-CRP, an indicator of systemic inflammation, may also associated with both urinary elements concentrations and serum albumin levels (36, 37). Therefore, we additionally adjusted for hs-CRP in the sensitivity analysis to explore its influence on the associations. In both stratified analysis and sensitivity analysis, urinary elements concentrations were incorporated into the models as continuous variables.

#### qgcomp analysis

Considering the uncertain effect directions of elements in the mixtures, we also induced qgcomp approach. The method estimates the change in serum albumin associated with a simultaneous quantile increase in all elements within the mixtures, while controlling for the same covariates in the GLM analysis. The weight of each element within the element mixtures was extracted to evaluate its relative influence on estimated associations between element mixtures and serum albumin. If the weights of elements were given different directions of effect, the weights were interpreted as the proportions of the positive (or negative) partial effect (i.e., the proportions of total effect for element mixtures), with weights in each direction summing to 1.0 (25).

#### BKMR analysis

To further consider the potential non-linearity and combined effects of element mixtures (24), we conducted a BKMR model using a kernel function to estimate the associations between element mixtures and serum albumin. The assumption of BKMR model included normal distribution of response variables (38). The same covariate set used in the GLM analysis was utilized for the BKMR analysis.The BKMR model is specified as follows:


$$\begin{array}{l} Y_{i}=h(Al_{i},\;As_{i},Ba_{i},Cd_{i}\;,\;Co_{i},\;Cr_{i},\;Cs_{i},\;Cu_{i},Fe_{i},Mn_{i}\;,N_{i}\\ Pb_{i},\;Se_{i},\;Sr_{i},\;Zn_{i})+\beta Z_{i}+e_{i} \end{array}$$

Where the Y_i_ represents the serum albumin for individual i; h() is an exposure-response function that can accommodate non-linear and non-additive dose-response relationships and interactions among elements in the mixtures; Z_i_ represents the covariates for individual i; β represents corresponding regression coefficients.

BKMR results were displayed as estimates of the (a) overall effect of the element mixtures; (b) univariate exposure–response relationships; (c) the single exposure-response functions for each element; and (d) bivariate exposure–response functions for pairs of elements.

## Results

### Participant characteristics

A summary of the participants' demographic data and clinical characteristics was presented in Table 1. Among the 275 participants included in the study, 153 (55.6 %) were female. The mean (SD) of age and BMI of the participants were 68.9 (6.8) years and 24.5 (3.8) kg/m^2^, respectively. Of these participants, 68.0% (*n* = 187) were never smokers, and 57.1% (*n* = 157) were never drinkers. The detection rates and distribution of 15 urinary elements concentrations are detailed in Table 2. For 15 elements excepting Mn, the proportions of samples having concentrations over the LOD were more than 99%.

**Table 1:** Demographic characteristics and health status of participants throughout the study period.

**Variables**	**Mean** ±**SD or** ***n*** **(%)**
Participants	275
Age, years	68.9 ± 6.8
BMI, kg/m^2^	24.5 ± 3.8
**Gender**	
Male	122 (44.4)
Female	153 (55.6)
**Education**	
Primary school or below	131 (47.6)
Middle school	55 (20.0)
High school or above	87 (31.6)
Refuse to answer	2 (0.7)
**Cigarette smoking**	
Never	187 (68.0)
Current	42 (15.3)
Former	41 (14.9)
Refuse to answer	5 (1.8)
**Alcohol consumption**	
Never	157 (57.1)
Current	21 (7.6)
Former	90 (32.7)
Refuse to answer	7 (2.5)
Serum albumin, g/L	46.2 ± 2.8

*BMI, body mass index; SD, standard deviation*.

**Table 2:** Detection rates and distribution for 15 adjusted urinary elements.

**Element (**μ**g/g**	**%**>**LOD**^a^	**GM(GSD)** ^b^	**Quartiles of adjusted urinary elements**			
**creatinine)**			**25th**	**50th**	**75th**	**90th**
Al	100.00	4.358 (2.144)	2.484	4.264	7.274	12.776
As	99.99	2.063 (2.200)	1.377	1.954	3.347	5.235
Ba	99.97	0.307 (3.424)	0.169	0.288	0.776	1.445
Cd	99.95	0.049 (3.295)	0.038	0.066	0.109	0.153
Co	99.99	0.104 (2.386)	0.051	0.105	0.212	0.338
Cr	100.00	0.274 (1.668)	0.189	0.267	0.381	0.569
Cs	100.00	0.947 (1.898)	0.716	0.966	1.324	1.724
Cu	99.99	1.082 (1.750)	0.749	1.093	1.601	2.118
Fe	100.00	3.903 (2.096)	2.170	3.850	6.474	10.753
Mn	61.80	0.031 (4.236)	0.008	0.027	0.094	0.235
Ni	100.00	0.311 (3.202)	0.188	0.375	0.736	1.077
Pb	99.86	0.118 (3.831)	0.042	0.141	0.325	0.631
Se	100.00	3.391 (2.153)	1.667	3.926	6.168	8.911
Sr	100.00	11.499 (1.909)	7.926	11.872	18.078	24.720
Zn	100.00	46.847 (1.776)	32.610	47.838	68.342	102.643

a *% > LOD, detection rate above limit of detection*.

b *GM, geometric mean; GSD, geometric standard deviation*.

*Al, aluminum; As, arsenic; Ba, barium; Cd, cadmium; Co, cobalt; Cr, chromium; Cs, cesium; Cu, copper; Fe, iron; Mn, manganese; Ni, nickel; Pb, lead; Se, selenium; Sr, strontium; Zn, zinc*.

### Associations of elements with serum albumin

#### GLM analysis

In this cross-sectional study of 275 elderly, we discovered that elements level were negatively associated with serum albumin after correction for multiple comparisons and adjustment for age, sex, BMI, educational attainment, cigarette smoking habits, alcohol consumption habits, hypertension status and diabetes status (Table 3). In detail, we observed elevated urinary concentrations as continuous variables of Al (−0.017, 95% CI: −0.026, −0.008), As (−0.011, 95% CI: −0.021, −0.002), Ba (−0.008, 95% CI: −0.014, −0.002), Co (−0.019, 95% CI: −0.028, −0.010), Cr (−0.023, 95% CI: −0.036, −0.010), Cu (−0.021, 95% CI: −0.032, −0.010), Fe (−0.018, 95% CI: −0.027, −0.008), Mn (−0.006, 95% CI: −0.011, −0.001), Se (−0.028, 95% CI: −0.038, −0.018), Sr (−0.020, 95% CI: −0.031, −0.009), Zn (−0.025, 95% CI: −0.038, −0.012) was associated with decreased serum albumin levels, respectively. These negative associations remained significant even with elements concentrations treated as categorical variables. In addition, we found negative associations between Cd, Cs, and serum albumin. In detail, compared with the lowest tertile, changes in serum albumin were −0.027 (95% CI: −0.047, −0.007) and −0.025 (95% CI: −0.046, −0.004) for the highest tertile of Cd and Cs.

**Table 3:** Associations between urinary elements and serum albumin analyzed by generalized linear regression models.

**Elements**	**Regression coefficients (95% CIs) by tertiles**			**Per 1-unit increase of each**	***p*** **-value** ^c^
	**of urinary metals concentrations** ^a^			**urinary metal** ^b^	
	**T1** ^e^	**T2** ^f^	**T3** ^g^		
Al	Ref^d^	−0.009 (−0.029, 0.011)	−0.032 (−0.052, −0.012)	−0.017 (−0.026, −0.008)	*p* <0.01
As	Ref	−0.005 (−0.025, 0.015)	−0.031 (−0.053, −0.009)	−0.011 (−0.021, −0.002)	0.02
Ba	Ref	−0.004 (−0.024, 0.015)	−0.029 (−0.049, −0.010)	−0.008 (−0.014, −0.002)	*p* <0.01
Cd	Ref	−0.018 (−0.038, 0.002)	−0.027 (−0.047, −0.007)	−0.006 (−0.013, 0.001)	0.08
Co	Ref	−0.034 (−0.054, −0.014)	−0.046 (−0.067, −0.025)	−0.019 (−0.028, −0.010)	*p* <0.01
Cr	Ref	−0.010 (−0.030, 0.010)	−0.028 (−0.048, −0.008)	−0.023 (−0.036, −0.010)	*p* <0.01
Cs	Ref	0.000 (−0.021, 0.021)	−0.025 (−0.046, −0.004)	−0.009 (−0.019, 0.002)	0.11
Cu	Ref	−0.004 (−0.024, 0.015)	−0.028 (−0.047, −0.008)	−0.021 (−0.032, −0.010)	*p* <0.01
Fe	Ref	−0.013 (−0.033, 0.006)	−0.037 (−0.057, −0.018)	−0.018 (−0.027, −0.008)	*p* <0.01
Mn	Ref	−0.013 (−0.033, 0.006)	−0.037 (−0.057, −0.017)	−0.006 (−0.011, −0.001)	0.02
Ni	Ref	−0.031 (−0.050, −0.011)	−0.002 (−0.023, 0.019)	−0.001 (−0.009, 0.006)	0.77
Pb	Ref	0.033 (0.012, 0.054)	−0.004 (−0.026, 0.018)	−0.003 (−0.009, 0.003)	0.33
Se	Ref	−0.031 (−0.052, −0.011)	−0.053 (−0.073, −0.033)	−0.028 (−0.038, −0.018)	*p* <0.01
Sr	Ref	−0.009 (−0.028, 0.011)	−0.037 (−0.057, −0.018)	−0.020 (−0.031, −0.009)	*p* <0.01
Zn	Ref	−0.002 (−0.022, 0.018)	−0.032 (−0.053, −0.011)	−0.025 (−0.038, −0.012)	*p* <0.01

*95% CIs, 95% confidence intervals; Al, aluminum; As, arsenic; Ba, barium; Cd, cadmium; Co, cobalt; Cr, chromium; Cs, cesium; Cu, copper; Fe, iron; Mn, manganese; Ni, nickel; Pb, lead; Se, selenium; Sr, strontium; Zn, zinc*.

*Boldface type indicates effect estimates were statistically significant, FDR corrected p < 0.05*.

*The model was adjusted for age, sex, BMI, educational attainment, cigarette smoking habits, alcohol consumption habits, hypertension status, and diabetes status*.

a *Regression coefficients and 95% confidence intervals of changes in serum albumin associated with the second tertile and the third tertile, with the first tertile as a reference*.

b *Regression coefficients and 95% confidence intervals of changes in serum albumin associated with continuous urinary elements concentrations*.

c *p-values were FDR corrected*.

d *Ref means reference*.

e *T1 means the first tertile*.

f *T2 means the second tertile*.

g *T3 means the third tertile*.

#### Stratified analysis and sensitivity analysis

We conducted analyses stratified by age, sex, cigarette smoking status, and alcohol consumption status (Supplementary Figure 1). The negative associations between As, Ba, Cd, Co, Cu, Fe, Mn, Se, Sr, Zn, and serum albumin did not differ significantly with age, sex, cigarette smoking status, or alcohol consumption status. However, a stronger negative association between Al and serum albumin (−0.026, 95% CI: −0.037, −0.015) was observed among females. In addition, a stronger negative association between Cr and serum albumin (−0.039, 95% CI: −0.056, −0.023) was observed among participants without alcohol consumption. Furthermore, we demonstrated that negative relationships of Cs with serum albumin (−0.021, 95% CI: −0.036, −0.007) among participants whose age was ≥65 years and those of Pb with serum albumin (−0.017, 95% CI: −0.029, −0.005) among participants aging <65 years. The results of sensitivity analyses additionally adjusting for hs-CRP yielded were unchanged, compared to the results of GLM analysis (Supplementary Figure 2).

### Associations of element mixtures with serum albumin

#### qgcomp analysis

**Figure 1:**
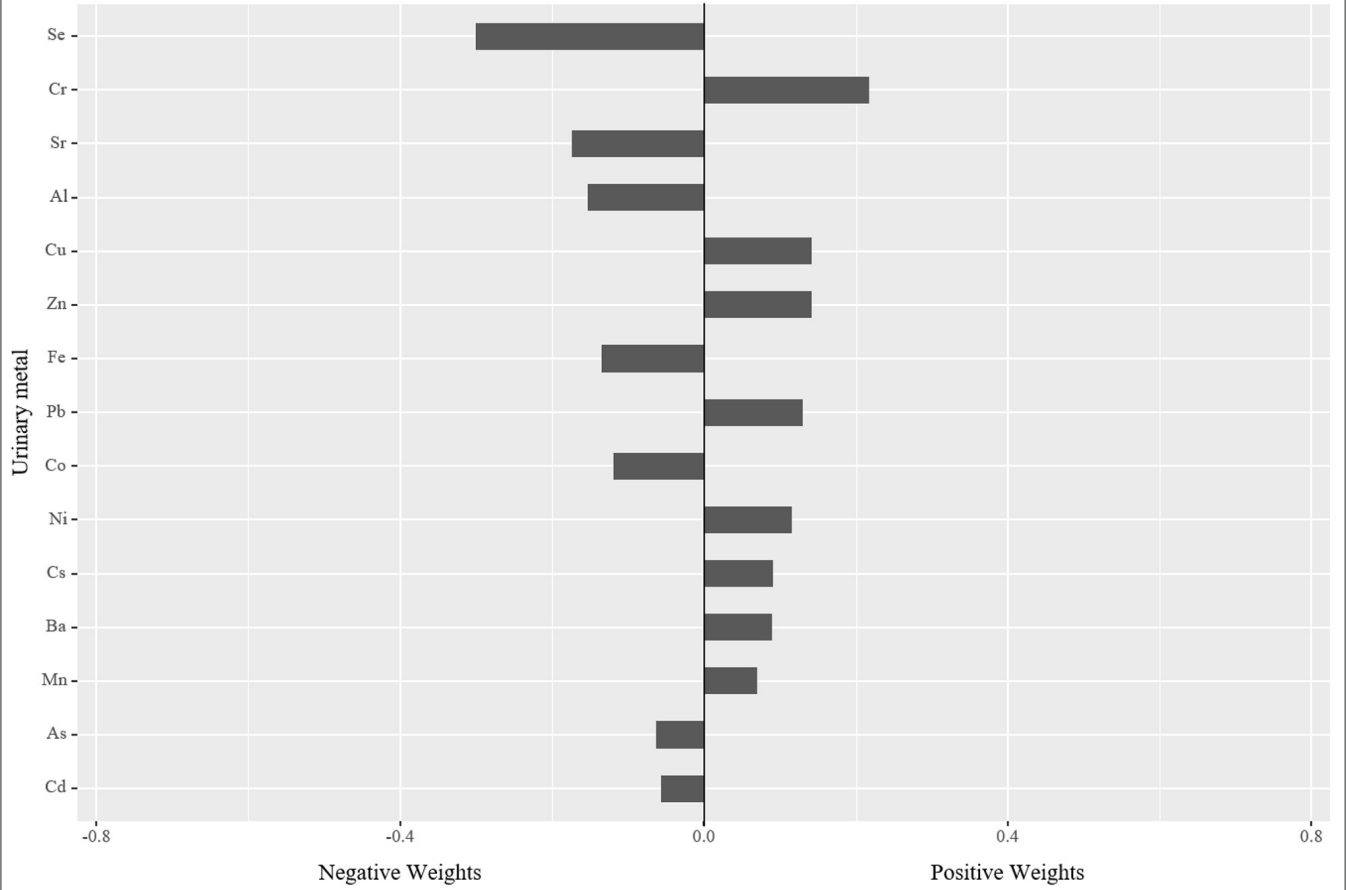
The qgcomp model regression index weights for serum albumin associated with element mixtures with adjustment for age, sex, BMI, educational attainment, cigarette smoking habits, alcohol consumption habits, hypertension status, and diabetes status. BMI, body mass index.

In the qgcomp mixtures approach (Figure 1), a negative association was observed between the element mixtures and serum albumin (−0.025, 95% CI: −0.040, −0.010). Urinary Cr (21.82%) concentration had the greatest positive contribution to the overall effect, and urinary Se (30.05%) concentration had the largest negative weight.

#### BKMR analysis

**Figure 2:**
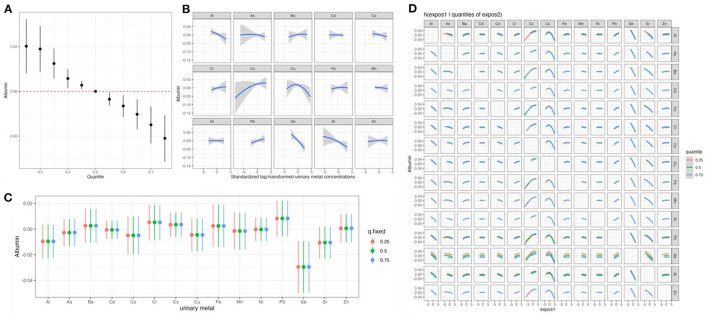
The effects of the element mixtures on serum albumin estimated by Bayesian Kernel Machine Regression. Model adjusted for age, sex, BMI, educational attainment, cigarette smoking habits, alcohol consumption habits, hypertension status, and diabetes status. **(A)** Overall effect of the element mixtures; **(B)** univariate exposure–response relationships; **(C)** the single exposure-response functions for each element; and **(D)** bivariate exposure–response functions for pairs of elements. Al, aluminum; As, arsenic; Ba, barium; Cd, cadmium; Co, cobalt; Cr, chromium; Cs, cesium; Cu, copper; Fe, iron; Mn, manganese; Ni, nickel; Pb, lead; Se, selenium; Sr, strontium; Zn, zinc; BMI, body mass index.

We first estimated changes in serum albumin associated with a concurrent increase or decrease in all 15 elements of mixtures from their median values (Figure 2). We found significant negative associations of the element mixtures with serum albumin, which was similar to the result of qgcomp analysis (Figure 2A). To explore potential non-linearity, we estimated single elements exposure-response functions when all other elements were fixed at their median values (Figure 2B). The exposure–response curves of elements suggested the negative trends of associations between Al, Co, Se, Sr, and serum albumin and positive trends of associations between Ba, Cr, Cs, Pb, and serum albumin. A converse *U*-shaped association between Cu and serum albumin was also observed (Figure 2B). The same models were utilized to estimate the changes in serum albumin associated with an interquartile range increase in each urinary element when the other elements were fixed at their 25th, 50th, or 75th percentiles (Figure 2C). An interquartile range increase in Se concentrations from the 25th to 75th percentile was associated with reductions in serum albumin of −0.03 (95% CI: −0.05, −0.01), −0.03 (95% CI: −0.05, −0.01), −0.03 (95% CI: −0.05, −0.01), when other elements were set at their 25th, 50th, and 75th percentiles, respectively (Figure 2C). Figure 2D showed a potential interaction between the 15 elements for serum albumin. The associations between each element and serum albumin did not differ by varying quantiles (25th, 50th, and 75th percentiles) of another element, when the other 13 elements in the mixtures were fixed at their median concentrations, indicating a lack of interaction between different elements.

## Discussion

### Interpretation of results

In this cross-sectional study of the elderly, we discovered that urinary essential and non-essential elements, including Al, As, Ba, Cd, Co, Cr, Cs, Cu, Fe, Mn, Se, Sr, Zn was negatively associated with serum albumin. In the qgcomp analysis, the element mixtures were negatively associated with serum albumin. Urinary Cr concentration had the greatest positive contribution to the overall effect, and urinary Se concentration had the largest negative weight. Similar to the results of the qgcomp analysis, a significant negative association between element mixtures and serum albumin was observed in the BKMR analysis. To our knowledge, this is the first report to comprehensively examine the relationship of essential and non-essential elements with serum albumin in the elderly. These findings provide new insights into how exposure to these elements may impact the nutrition status of the elderly.

The results in GLM, qcomp, and BKMR analyses were compared in Supplementary Table 1. In the GLM analysis, we found that urinary element as the continuous variable (Al, As, Ba, Co, Cr, Cu, Fe, Mn, Se, Sr, Zn) was negatively associated with serum albumin, respectively. The negative overall effects of element mixtures on serum albumin were also found in the qcomp and BKMR analyses. Further, over half of the elements in the mixtures (Al, As, Cd, Co, Fe, Pb, Se, Sr) were found to have the same direction or trend of associations with serum albumin in the three analyses. However, we also found that the directions or trends of associations between Ba, Cr, Cs, Cu, Mn, Ni, Zn, and serum albumin were inconsistent in the three analyses, and these inconsistencies can be explained by model assumptions and modeling strategies. The model assumptions of the three analyses were different. The model assumptions of the GLM and qgcomp in the present study included linear associations between exposure variables and outcome variables and the normal distribution of the outcome variables. In contrast, BKMR only requires outcome variables to be normally distributed (25, 39). Meanwhile, the modeling strategies of the three analyses were distinct (38). First, if 15 elements were considered simultaneously in the model of a small sample study (*N* = 275), the model could be over-fitted, additionally considering potential non-linear and non-additive associations. Therefore, we included each element in the GLM model to estimate the changes in serum albumin levels associated with each element, respectively. Second, we utilized the qgcomp method to estimate the change in serum albumin associated with a simultaneous quantile change in all 15 elements within the element mixtures, considering the collinearity and the difference in the direction of the effect of the elements within the element mixtures. Finally, the BKMR with a kernel function was adapted to estimate the association between element mixtures and serum albumin, considering high-dimensional, collinearity among elements and potential non-linear and non-additive associations. Hence, it is suggested that future research should consider the assumptions and characteristics of multiple models, and use multiple statistical analyses to explore the associations between urinary elements and serum albumin.

To date, only a few prior epidemiologic studies have evaluated the relationships between elements and serum albumin. Similarly, a cross-sectional study of 240 male tannery workers reported that prolonged exposure to hexavalent Cr (Mean: 4.89 μg/L in smokers) is likely to reduce serum albumin levels (21). Another cross-sectional study of 1,171 participants living in northeast China demonstrated that negative association between urinary Cr (Median: 4.41 μg/L) and serum albumin (18). The biological mechanisms explaining associations between urinary elements and serum albumin are not well-understood. Impaired liver function and disrupted serum albumin transportation involved in the pathogenesis of low serum albumin attributed to essential and non-essential elements. First, the liver both synthesizes albumin and acts as the central organ for the metabolism of elements (40, 41). The elements dyshomeostasis stimulate oxidative stress, mitochondrial damage, and inflammatory responses which leads to histopathological damage and apoptosis of liver cells. Furthermore, impaired liver function inhibits albumin synthesis, further reducing serum albumin levels. As a non-essential element, Cr(VI) targeted and inhibited mitochondrial respiratory chain complex I and III to induce electron transfer chain dysfunction, which played an important role in Cr(VI)-induced cytotoxicity in the human L-02 hepatocyte line (42). Impaired liver function was also observed in dyshomeostasis of essential elements. The elevation of liver dysfunction biomarkers (aspartate transaminase, alanine transaminase, and bilirubin), and cellular degeneration and necrosis of hepatocytes were observed in male Wistar rats which were exposed orally to Mn chloride (20 mg/mL) for 30 days (43). Furthermore, accumulating evidence shows that serum albumin levels were low in patients with advanced and decompensated cirrhosis, due to a reduction in the hepatocyte mass (44). Second, serum albumin could bind and carry hormones, enzymes, and elements such as Co, Cr, and Ni throughout the body. Hence, elements directly affect serum albumin and reduce serum albumin status. Experimental studies have suggested that Co, Cr, Ni induce aggregation synergistically of serum albumin and cause low serum albumin levels. Future epidemiological and experimental studies are needed to validate results of our study and elucidate underlying biological mechanisms.

Serum albumin is a significant indicator of nutrition status in the elderly, and malnutrition characterized by low serum albumin was independently associated with increased adverse cardiovascular events and mortality (9, 45). In the present study, we demonstrated that elevated essential and non-essential elements concentrations were associated with decreased serum albumin levels in the elderly. In another analysis of the same population, we found that essential and non-essential elements, including Zn, Sr, Cd, and element mixtures disturb glucose levels (28). Meanwhile, another parallel evidence shows urinary Cu concentration was strongly positively associated with urinary albumin/creatinine ratio, which was a sensitive indicator of chronic kidney disease (CKD) (27). The decreased renal function associated with elevated Cu concentrations was also observed in the three-wave repeated-measures study of 201 older adults (46). Impaired glucose levels and elevated risk of CKD were reported to be continuous risk factors for cardiovascular disease (47, 48). It is becoming increasingly evident that chronic long-term exposure to essential and non-essential elements leads to a multipronged assault to many organ systems in an insidious but relentless manner. Furthermore, a systematic review and meta-analysis of 37 unique epidemiological studies comprising 348,259 participants support that elements including As, Pb, Cd, and Cu are associated with the increased risks of cardiovascular disease and coronary heart disease (49). Malnutrition intervention has been reported to improve these chronic disease statuses (50, 51). In combination with these results, it was suggested that elevated essential and non-essential elements levels may increase the potential risk of cardiovascular disease in the elderly by promoting malnutrition. Formal mediation analysis using effect decomposition may help elucidate the role of malnutrition as a mediator, but much work remains at elucidating mechanisms before understanding the potential bias of such approaches (52). Our results support the need for additional interventions to reduce element exposures among the elderly because these exposures may modulate nutrition status and risk of cardiovascular disease.

### Limitations and strengths

This study had several limitations. First, the participants in our study were elderly participants (age ≥ 60 years) residing in Beijing, China. Thus, the generalisability of the results to more general populations may be limited. Second, we used questionnaires to collect information regarding the participants' alcohol consumption and cigarette smoking habits. Consequently, unmeasured confounding factors and recall bias is unavoidable. Third, the cross-sectional study design inhibited inference of causal relationships, and large-scale cohort studies were warranted to verify the results of our study. Fourth, considering the limited source, we did not collect dietary information. Specific dietary pattern, particularly dietary protein intake, was widely recognized as a significant confounder of associations between urinary elements and serum albumin. The intake of high protein foods (i.e., seafood products) contaminated with heavy metals affects internal burden levels of metals (53). Meanwhile, it was reported that animal protein intake was related to serum albumin levels, while vegetable protein intake was not related (54). The participants in our study were community-dwelling elderly (above 60 years or more). The dietary patterns of these elderly were characterized by excessive grain intake and inadequate animal protein intake and were relatively stable (55). Meanwhile, the study was conducted during the winter (November–December). Therefore, the effect of individual and seasonal-related dietary patterns, especially for dietary protein on the association was minimized. However, it is still emphasized the need for dietary protein intake measurements in other epidemiological studies exploring associations between elements and serum albumin. Lastly, we selected urinary elements as the elements biomarkers to investigate, but serum or whole blood selenium may have been more favorable alternatives. However, there was no gold standard regarding the most appropriate matrix of all 15 metals due to the significant difference in metal metabolism in the body (56, 57). Urine was considered to be a non-invasive, accessible, and more stable biomarker for metal mixtures than whole blood and serum. Hence, urine is the most commonly collected matrices in human biomonitoring (58). Furthermore, we corrected concentrations of urinary elements by urinary creatinine to control for the effect of urine dilution and increase the accuracy of exposure assessment in the present study. Further studies need to select the most sensitive, reliable, or stable biomarker of each metal exposure to explore associations between elements and serum albumin. Despite these limitations, several strengths should also be considered. To our knowledge, this is the first study to comprehensively evaluate the associations of essential and non-essential elements with serum albumin. Second, we adopted three different statistical methods, including GLM, qgcomp and BKMR to verify the stability of the results. Finally, our findings provide clues to explore the involvement of nutrition status in the associations between essential and non-essential elements and cardiovascular disease among the elderly.

## Conclusion

We discovered that essential and non-essential elements were negatively associated with serum albumin in the elderly. This indicates that essential and non-essential elements have an adverse effect on nutrition status among the elderly. In the future, large-scale cohort studies among the general population are required to verify the associations of essential and non-essential elements with serum albumin, and determine the relevant underlying mechanisms.

## Data availability statement

The original contributions presented in the study are included in the article/Supplementary Material, further inquiries can be directed to the corresponding author.

## Ethics statement

The studies involving human participants were reviewed and approved by the Institutional Ethics Committees of the Institute of Basic Medicine in the Chinese Academy of Medical Sciences. The patients/participants provided their written informed consent to participate in this study.

## Author contributions

AL: conceptualization, methodology, data-analysis & interpretation, writing-original draft, writing-review & editing, and funding acquisition. QZ: data-cleaning & interpretation, writing-original draft, and writing-review & editing. JZ, LL, YM, MZ, JX, and XG: investigation and writing-review & editing. QX: funding acquisition, writing-review & editing, and supervision. All authors contributed to the article and approved the submitted version.

## Funding

This study was supported by the China Medical Board (Grant No. 15-230), the Fundamental Research Funds for the Central Universities (Grant No. 3332019147), Peking Union Medical College Graduate Innovation Fund (Grant No. 2019-1004-02), Peking Union Medical College Graduate Innovation Fund (Grant No. 2022xscx-02), the China Prospective cohort study of Air pollution and health effects in Typical areas (C-PAT) (Grant No. MEE-EH-20190802), and the Chinese Academy of Medical Science Innovation Fund for Medical Sciences (Grant No. 2017-I2M-1-009). These funders did not participate in the organization of the study design, data collection, analysis, or writing and did not impose any restrictions regarding the publication of the report.

## Conflict of interest

The authors declare that the research was conducted in the absence of any commercial or financial relationships that could be construed as a potential conflict of interest.

## Publisher's note

All claims expressed in this article are solely those of the authors and do not necessarily represent those of their affiliated organizations, or those of the publisher, the editors and the reviewers. Any product that may be evaluated in this article, or claim that may be made by its manufacturer, is not guaranteed or endorsed by the publisher.
