# Supplementary material for: The effect of urinary essential and non-essential elements on serum albumin: Evidence from a community-based study of the elderly in Beijing
Source: Front Nutr. 2022 Jul 18;9:946245. doi: 10.3389/fnut.2022.946245 (PMC9342688; doi:10.3389/fnut.2022.946245)
Supplement: Supplementary file 1 [file Data_Sheet_1.pdf]

Supplementary Materials

The effect of urinary essential and non-essential elements on serum albumin: Evidence from a community-based study of the elderly in Beijing

Ang Li[1,2†], Quan Zhou[1,2†], Yayuan Mei[1,2], Jiaxin Zhao[1,2], Liu Liu[3], Meiduo Zhao[1,2], Jing Xu[1,2], Xiaoyu Ge[1,2], Qun Xu[1,2*]

[1]Department of Epidemiology and Biostatistics, Institute of Basic Medical Sciences Chinese Academy of Medical Sciences, School of Basic Medicine Peking Union Medical College, Beijing 100005, China

[2]Center of Environmental and Health Sciences, Chinese Academy of Medical Sciences, Peking Union Medical College, Beijing 100005, China

[3]Chaoyang District Center for Disease Control and Prevention, Beijing, 100021, China

* Correspondence to:

Qun Xu, PhD

Department of Epidemiology and Biostatistics, Institute of Basic Medical Sciences Chinese Academy of Medical Sciences, School of Basic Medicine Peking Union Medical College

Beijing 100005, China

Tel: +86 10 69156403

Fax: +86 10 69156403

E-mail: xuqun@ibms.cams.cn

[†]These authors are co-first authors who contributed equally to this paper.

Catalogue

Figure S1. Estimates and 95% confidence intervals of serum albumin associated with one-unit increase of log-transformed urinary elements concentrations, stratified by sex, age, cigarette smoking habit, and alcohol consumption habit. 3
Figure S2. Estimates and 95% confidence intervals of serum albumin associated with one-unit increase of log-transformed urinary elements concentrations, additionally adjusting for high-sensitivity C-reactive protein on the basis of GLM analysis. 4
Table S1. Summary of results in the three models 5

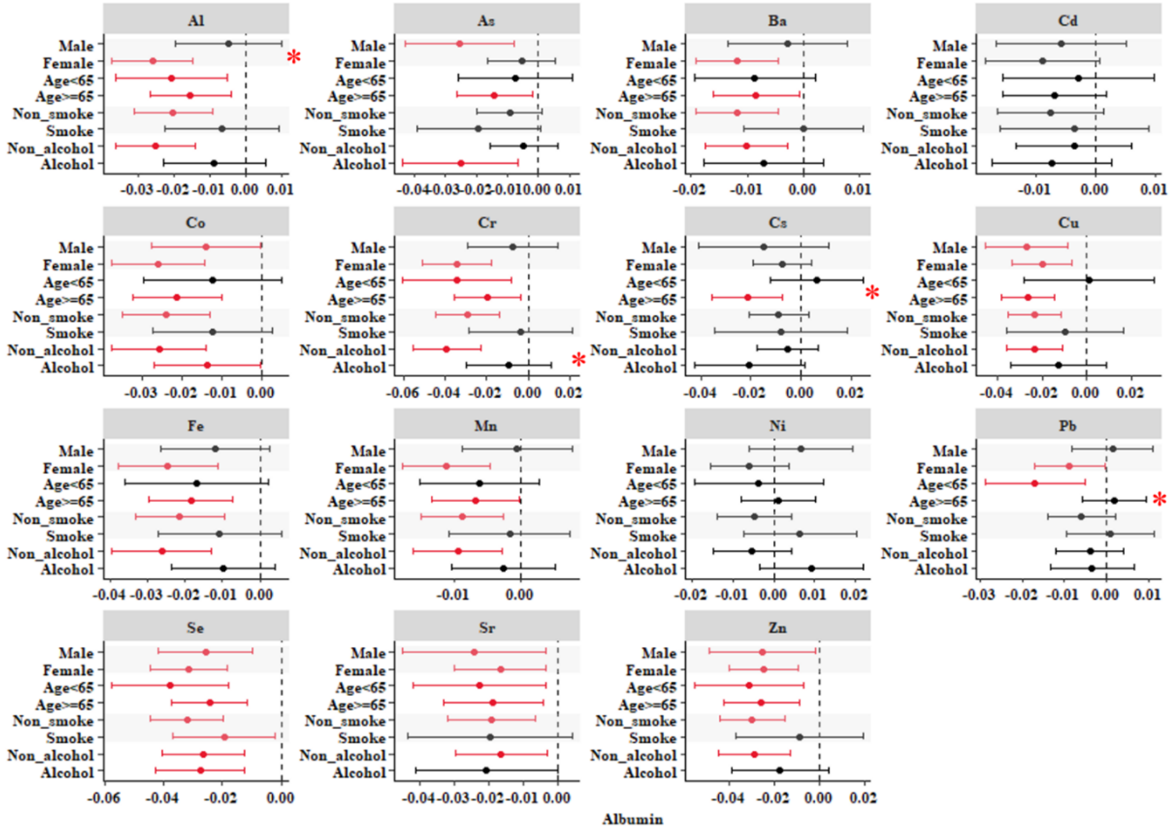

Figure S1. Estimates and 95% confidence intervals of serum albumin associated with one-unit increase of log-transformed urinary elements concentrations, stratified by sex, age, cigarette smoking habit, and alcohol consumption habit.

Red indicates statistical significance ($p < 0.05$). Asterisk (*) indicates significant difference in subgroups.

Abbreviation: Al: aluminum; As: arsenic; Ba: barium; Cd: cadmium; Co: cobalt; Cr: chromium; Cs: cesium; Cu: copper; Fe: iron; Mn: manganese; Ni: nickel; Pb: lead; Se: selenium; Sr: strontium; Zn: zinc.

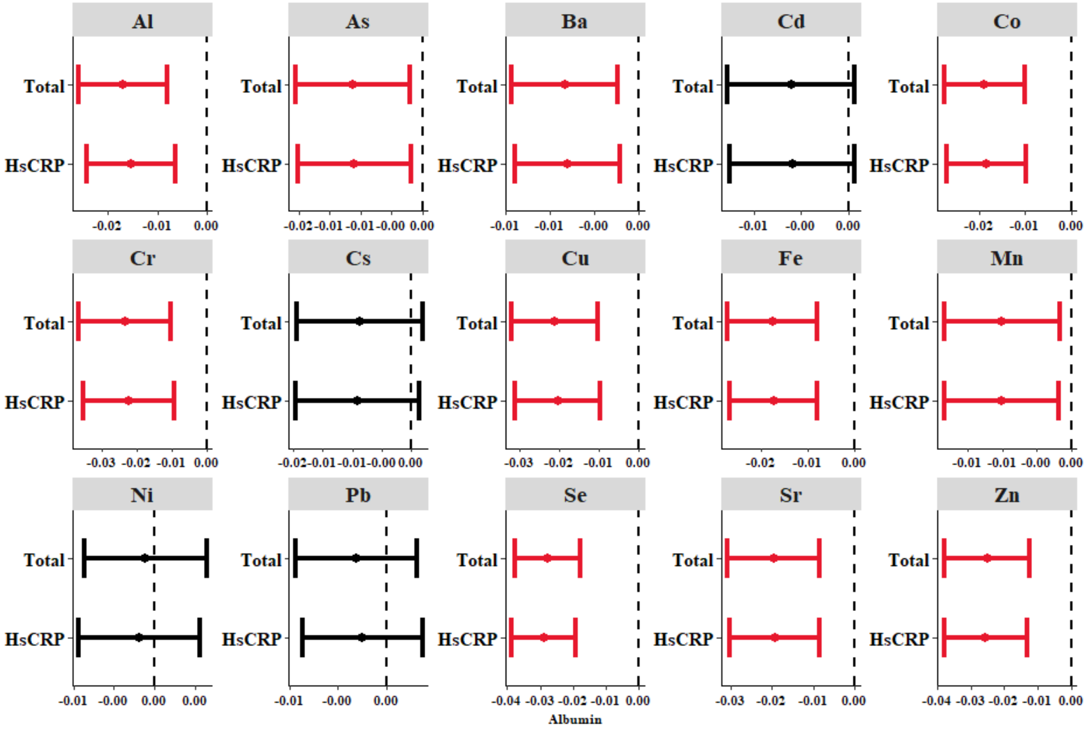

Figure S2. Estimates and 95% confidence intervals of serum albumin associated with one-unit increase of log-transformed urinary elements concentrations, additionally adjusting for high-sensitivity C-reactive protein on the basis of GLM analysis.

The total means the results of the GLM analysis.

Red indicates statistical significance ($p < 0.05$).

Abbreviation: Al: aluminum; As: arsenic; Ba: barium; Cd: cadmium; Co: cobalt; Cr: chromium; Cs: cesium; Cu: copper; Fe: iron; Mn: manganese; Ni: nickel; Pb: lead; Se: selenium; Sr: strontium; Zn: zinc; Hs-CRP: high-sensitivity C-reactive protein.

Table S1. Summary of results in the three models

	GLM	qgcomp	BKMR
Mixture	-	negative association	negative association
Elements			
Al	negative association	negative weight	negative trend
As	negative association	negative weight	-
Ba	negative association	positive weight	positive trend
Cd	-	negative weight	-
Co	negative association	negative weight	negative trend
Cr	negative association	positive weight	positive trend
Cs	-	positive weight	positive trend
Cu	negative association	positive weight	converse-U trend
Fe	negative association	negative weight	-
Mn	negative association	positive weight	-
Ni	-	positive weight	-
Pb	-	positive weight	positive trend
Se	negative association	negative weight	negative trend
Sr	negative association	negative weight	negative trend
Zn	negative association	positive weight	-

Note: - means no association, weights or trends were observed.
